# Finite element method for estimation of applanation force and to study the influence of intraocular pressure of eye on tonometry

**DOI:** 10.1007/s10792-021-02157-6

**Published:** 2022-06-04

**Authors:** R. B. Bharathi, Rakshath G. Poojary, Gopalakrishna K. Prabhu, Ramesh S. Ve

**Affiliations:** 1 Department of Electrical and Electronics Engineering, Manipal Institute of Technology, Manipal Academy of Higher Education, Manipal, India; 2 Department of Mechanical and Manufacturing Engineering, Manipal Institute of Technology, Manipal Academy of Higher Education, Manipal, India; 3 Department of Electronics and Communication Engineering, Manipal Academy of Higher Education, Manipal University Jaipur, Jaipur, India; 4 Department of Optometry, Manipal College of Health Professions, Manipal Academy of Higher Education, Manipal, India

**Keywords:** Biomechanical simulation, Finite element analysis, Tonometry, Glaucoma, IOP

## Abstract

**Purpose:**

Discover the associations of force of applanation on the eye with the plunging depth of the cornea and quantify them. The results will be utilized as the feedback parameter in the new prototype development of eye care instruments as additional force may damage the internal structure of the eye or may result in erroneous output.

**Method:**

A finite element-based eye model is designed utilizing the actual dimensions of the human eye. A standardized tonometer is designed and the simulation is carried out at predetermined deformation of the cornea to find the force of applanation on the cornea during tonometry. Adding on, the influence of IOP during tonometry is analyzed for a range of plunging depths of the cornea.

**Results:**

The graphical results inferred the linear relation between the force of applanation with the deformation of the cornea and the results are quantified. The resulting deformation and stress plot of FEM based simulation approach is analyzed and observations regarding deformations and stress are made.

**Conclusion:**

The human eye is successfully developed and also computed force on the cornea during tonometry is validated. The inference drawn from the deformation plot and stress plot is that the junction of cornea–sclera along with cornea-tonometer periphery undergo maximum deformation and experiences the highest stress compared to other areas of the eye while during tonometry.

## Introduction

Glaucoma refers to a group of diseases that damages the optic nerve. It is a pathological condition that causes optic nerve neuropathy and structural damage to the eye. This finally culminates in permanent vision loss [1]. There is no cure for the disease at later stages, diagnosis of the disease at an early stage is very important to slow down its progress [2]. It is also the second leading cause of blindness after cataracts. Elevated intraocular pressure (IOP) is one of the main risk factors in glaucoma. IOP is important in maintaining the structure and function of the eye [3]. The rise in IOP is due to reduced aqueous humor outflow at the trabecular meshwork which in turn changes the outflow resistance [4]. The study done in [5] summarises the relationship between the aqueous humor outflow resistance and intraocular pressure.

The present study is focused on the biomechanical FEM (finite element model) simulation of an asymmetrical 3-D model of the human eye constructed consisting of cornea and sclera under tonometry. The study is also evolved with the design of a standard tonometer which consists of a probe to applanate the cornea. Currently, Pneumotonometer, tonopen, Mackay Marg tonometer, and Icare tonometer are the existing tonometers that utilize the plunger or probe to measure the IOP of the eye. A plunger with a 5 mm diameter inside the Pneumotonometer placed on the eye for 5–10 s will measure the pressure inside the eye for the given displacement [6]. Tonopen incorporates a similar principle to that of Mackay and Marg tonometer [6]. It has several additional features such as small, handheld, battery-powered, and incorporates an internal chip to store the data. The works of literature [7–10] describe the probe and the characteristics of a standard Icare tonometer. Icare tonometer works on the principle of rebound tonometry. A plastic ball on a stainless-steel wire is a probe of 1.8 mm diameter which momentarily touches the cornea to measure the IOP [6].

The explicit dynamic analysis was conducted for the blunt impact of foreign bodies on the eye [11]. The eye model is resting in the orbital muscle and fat in frictionless contact. The model of the eye was considered as hollow. A similar model was created but the fluid part of the model, Aqueous humor was modeled as liquid with shock EOS linear *C*_1_ = 1530 m/s, *s*_1_ = 2.1057 and vitreous humor as viscoelastic *G*_0_ = 10 Pa, *G*_∞_ = 0.3 Pa, *β* = 14.26 1/s, *k* = 2.0 GPa, sclera and cornea were modeled as the nonlinear stress–strain as material properties [12]. The viscoelastic model of the eye was created for the blunt impact of the eye [13]. The eye model was considered viscoelastic based on soft fiber-reinforced composites [14]. The material properties of the eye tissue were calculated by conducting materials testing [15]. However, the properties of the material were calculated in vitro conditions. The eye model was considered as rigid with vitreous as a solid mass with hydrostatic pressure as 20 mmHg (2.66645 kPa), i.e., intraocular pressure, atmospheric pressure was set at the anterior. Similarly, biomechanical properties of the cornea will influence the reading of tonometry and have been concluded that the rigid corneas will result in higher IOP value in tonometry [16].

In the present research, FEM-based evaluation is used to calculate the amount of force exerted by the tonometer on the cornea during tonometry with a standard tonometer. The FEM study incorporates a tonometer cylindrical probe of 1.7 mm diameter [6]. Predetermined force on the eye using a probe or an indenting element is also an important factor for determining the IOP of the eye. Similarly, excess force on the eye may also damage the internal structure of the eye. Hence, quantification of force on the cornea becomes very much essential during tonometry with a standard tonometer. Meanwhile, variation in the IOP of the eye may affect the reading of force on the cornea during the clinical procedure tonometry. Thus, the analysis is also focused on finding the influence of IOP on the amount of force applied to the cornea during tonometry. The response of the eye may change due to external factors such as disease, surgery, and injury and will influence the visual performance of the eye [17]. Hence, deformation and the stress analysis of cornea during the tonometry test are investigated in the simulation study.

The tabulated results from this study such as force, stress, and deformation can be implemented in the new technology development of eye care instruments such as a tonometer, goniolens, pachymeter and hence can be utilized in the eye care industry.

The designed model of the human eye is close to the actual dimensions of the eye and hence the model is close to the actual human eye. The investigation of the force on the cornea during eye care testing procedure is not addressed in clinical and industrial side and hence the study about the applanation force during eye care testing procedure is unique.

## Materials and methods

### Modelling of eye geometry

**Fig. 1:**
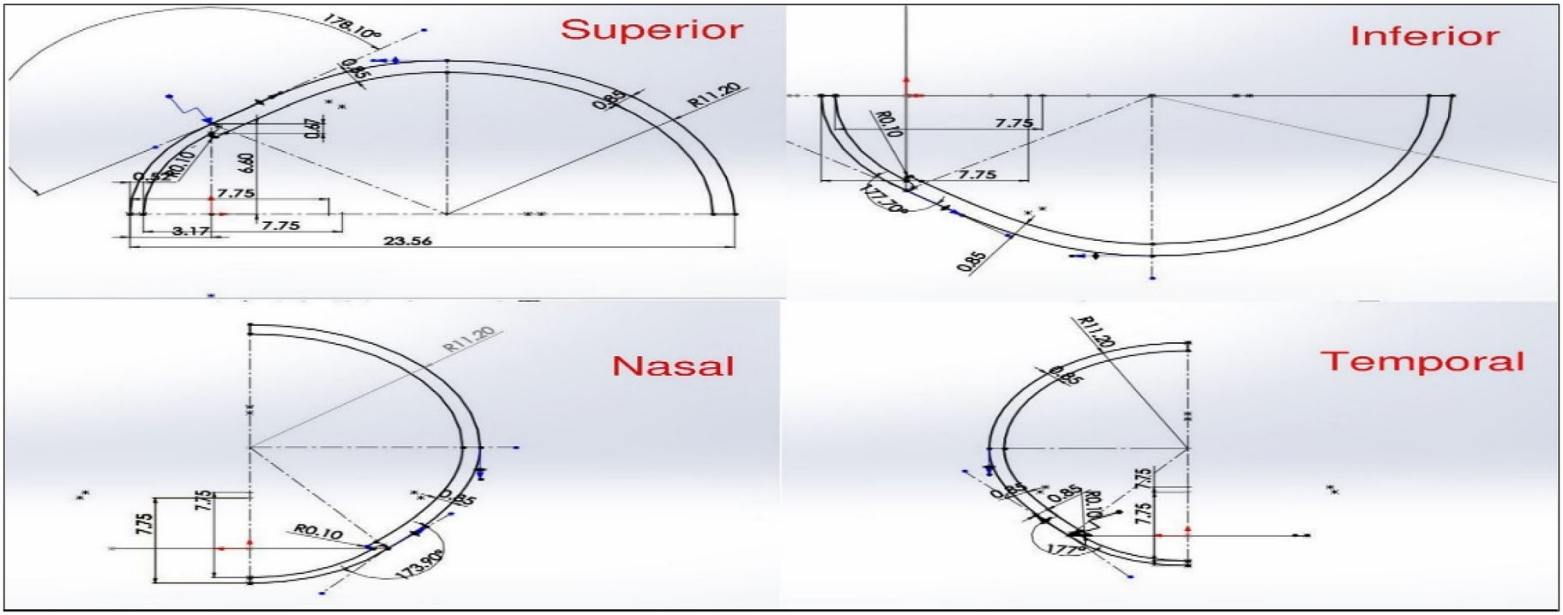
Key point sketches (dimensions in mm)

The eye model was made with the loft protrusion method. The dimensions of different parts of the eye model are listed in Table 1 and are referred to from the references [18–26]. Initially, the cornea base was created with an ellipse and it was used as the guide curve for loft protrusion in the eye model. The eye model was assumed to have been thin shell-like structure and the 2d cross section of the eye was created at the four key points. The regions used were superior, inferior, temporal, and nasal (Fig. 1). This method of modeling was chosen to get approximate asymmetry in the eye. The internal parts of the eye were not modeled because the pressure at any point inside a closed system with static liquid is constant [27]. Hence, modeling the internal parts will not make much difference in terms of the pressure changes.

**Table 1:** Eye modeling parameters

Ocular parameters		References
Corneal anterior curvature	7.75 mm	[18]
*Corneal thickness*		
Central	0.52 mm	[19, 20]
Peripheral	0.67 mm	[19, 20]
Scleral radius	11.2 mm	[22]
Subfoveal scleral thickness	317 µm	[23]
Choroidal thickness	298 µm	[24]
Axial length	22.7 mm	[25]
*Corneoscleral junction angle*		
Superior	178.1°	[26]
Inferior	177.7°	[26]
Nasal	173.9°	[26]
Temporal	177.0°	[26]

### Analysis setup

The eye model was divided into nine different parts as 4 parts of the sclera, 1 part of the cornea, and 4 parts of the corneoscleral junction for the simplification of the meshing. The average element size of the complete eye model is 1.9816 mm. The mesh model resulted in a total node count of 58,999 and a total element count of 11,613.

The main aim of the simulation is to determine the force applied by the tonometer over the eye. The tonometer will apply force over the surface of the cornea to provide a predetermined deformation or plunging depth on the surface of the cornea. In the previous simulation in the literature, the main concentration was impact analysis. Since the tonometer is not impacted on the eye, analysis was carried out in ANSYS static structural to find the reaction force or applanation force responsible for deformation of the cornea for a given fixed set of displacement of the tonometer. This is simulated by moving the tonometer probe onto the eye in direction towards the retina or of the apex of the orbit and calculating the reaction force on the tonometer. The tonometer was taken as a cylinder with a diameter of 1.7 mm [6]. The tonometer was modeled and made in contact with the cornea such that there is no penetration and deformation. This contact is defined as frictionless.

Human ocular tissues are generally viscoelastic and exhibit nonlinear material properties [28]. But it is difficult to incorporate the nonlinear properties into the eye mode [29]. Hence, the material properties of the eye model were assumed to be homogenous, isotropic, and linearly elastic. Table 2 lists the properties of the different parts of the eye model and are referred from [30, 31].

**Table 2:** Different material properties corresponding to different parts of the eyeball model

Material properties/part of the eyeball	Young’s modulus	Poisson’s ratio	Bulk modulus	Shear modulus (MPa)	Density (kg/mm^3^)
Sclera	2 MPa	0.4	3.3333 MPa	0.71429	9.6e–07
Cornea	0.2 MPa	0.43	0.47619 MPa	0.06993	9.6e–07
Tonometer probe	2e+05 MPa	0.3	1.6667e+05 MPa	76,923	7.5e–06

The sclera was given the condition of the remote displacement zero. This boundary condition allows the deformation to occur on the mesh elements of the eye but constraints the movement in the space. This simulates the eye held in place by the eye socket which is similar to the research done in [11, 15] but without modeling of the orbital muscles. The intraocular pressure inside the eye is fixed normal to the sclera and cornea surface this pressure diverges outward. The tonometer probe is constrained to move in a positive X direction, i.e., from the cornea center toward the sclera such that the tonometer contacts the surface of the cornea and will deform the cornea. In the study, displacement of the tonometer varies from 0.3 mm-0.7 mm and the IOP eye is varied in the normal range from 10 to 20 mmHg during which the behavior of the eye is studied and analyzed.

## Results

The human eye is modeled using FEM-based simulation software that is more close to the actual size. The model consists of the human cornea, sclera (4 components), and the junction between cornea and sclera. The tonometer probe designed is 1.7 mm in diameter. The simulation is carried out on the designed model of the eye, by placing the standardized designed tonometer at the center of the cornea. The deformation of the cornea takes place when external pressure exceeds the internal pressure [15], and a set of the predetermined plunging depth of the cornea (0.3–0.7 mm) is produced. The reaction force responsible for the known deformation at the center of the cornea is tabulated and analyzed. To analyze the effect of varying IOP of the eye on tonometry, the research is repeated in the normal range of IOP (10–20 mm Hg).

**Fig. 2:**
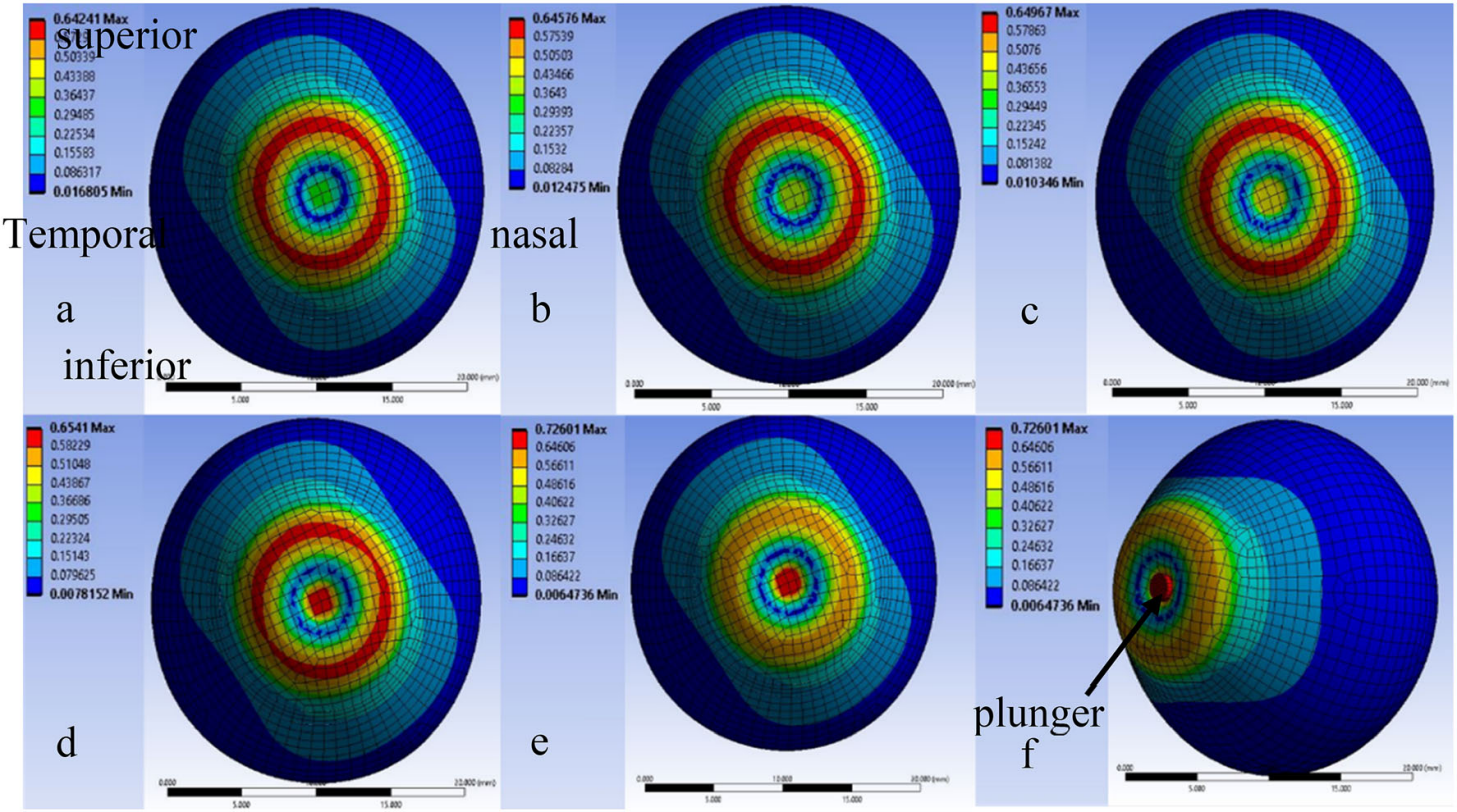
Deformation of cornea under tonometry for varying displacement of tonometer, **a** 0.3 mm, **b** 0.4 mm, **c** 0.5 mm, **d** 0.6 mm, **e** 0.7 mm, **f** 0.7 mm isometric view with tonometer with 20 mmHg of IOP

**Fig. 3:**
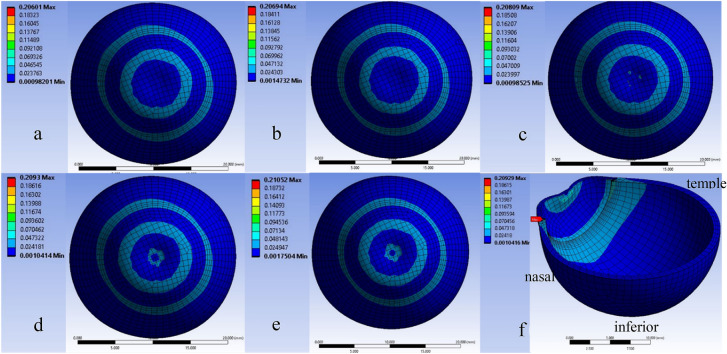
Stress distribution (MegaPascal) of eye under tonometry for 20 mmHg IOP for displacement of **a** 0.3 mm, **b** 0.4 mm, **c** 0.5 mm, **d** 0.6 mm, **e** 0.7 mm, **f** 0.6 mm cut section view

The FEM-based investigation has resulted in a deformation chart of the eye and also stress distribution of the eye during tonometry. Figure 2 indicates the deformation chart and Fig. 3 is the stress distribution chart of the eye with plunging depth of cornea varied from 0.3 to 0.7 mm at the center of the cornea and the IOP of eye maintained at 20 mmHg. The chart indicates the variation in the magnitude of deformation and stress on the surface of the cornea, sclera, and the junction of cornea and sclera. The force on the cornea is plotted against the deformation in Plot 1. The magnitude of applanation force on the cornea during tonometry is investigated for a predetermined set of deformation of the cornea and is tabulated in plot 1. Plot 1 also depicts the influence of IOP on tonometry.

**Plot 1:**
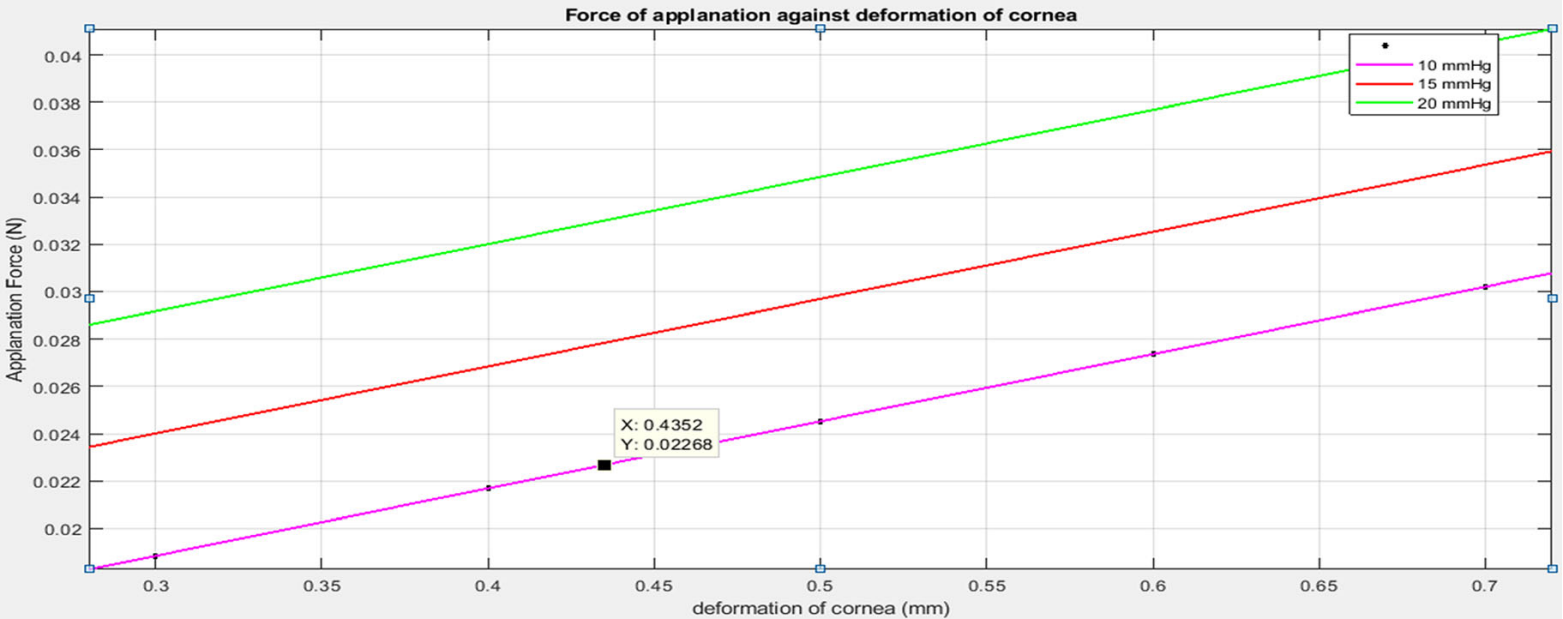
Force (Newton) exerted on cornea by tonometer for varying deformation of cornea (mm)

## Discussion

The eye model with dimensions obtained from [18–26] constituting the cornea, sclera, and junction of cornea and sclera was modeled using FEM-based simulation software. The displacement of the cornea during tonometry is considered in the range of 0.3–0.7 mm along the direction from the cornea toward the retina. The study is carried out at the normal range of IOP of the human eye from 10 to 20 mmHg in steps of 5 mmHg.

The deformation chart of the human eye during tonometry is indicated in Fig. 2. The cornea at the center is deformed by a tonometer (indicated by an arrow). The chart indicates that the junction of cornea and tonometer undergo maximum deformation compared to plunging depth and the deformation is in outwards expansion. This is due to the material properties of the cornea which is less stiff compared to the sclera and the sclera is held in place which results in more deformation at the junction of the cornea tonometer periphery. Meanwhile, the human eye model is more close to the actual eye and is not symmetrical, and superior-temporal zone and nasal-inferior zone sections at the junction of the cornea and sclera are thinner compared to the other parts of the sclera. This results in the eye deforming more at the junction of the cornea and sclera regions. The deformation chart also depicts the behavior of the eye during tonometry. For the plunging depth from 0.3 to 0.5 mm the maximum deformation is under 0.65 mm and as the plunging depth increases the maximum deformation remains at 0.75 mm. This means during tonometry the eye will undergo deformation in vertical elongation.

Figure 3 summarizes that the stress is concentrated at the junction of cornea and sclera and experiences maximum stress of about 0.2 MPa during 0.3–0.7 mm displacement of the cornea for constant IOP of the eye. Figure 3f points at the position of maximum stress at the junction of cornea and sclera. It also depicts that along with the junction, the front side of the eye is under stress during tonometry. The literature [29] reveals that it would be highly challenging to evaluate the stress concentration at the junction during tonometry.

The simulation is carried out for determining the amount of force applied on the cornea for the predetermined displacement of the cornea during the tonometry test. Plot 1 demonstrates that the force applied on the cornea is small at lower deformation of the cornea and further steeply increases at larger deformation of cornea. Hence, the force increases from 0.02 to 0.045 N for an increase in the plunging depth of the cornea from 0.3 to 0.7 mm. The force on the cornea increases with increasing the IOP of the eye from 10 to 20 mmHg during tonometry. Plot 1 concludes that force on the cornea is increased by approximately 0.004 N for every 5 mmHg increase in IOP of the eye.

The research work is carried out with a design of a standard tonometer of size 1.7 mm diameter and also by considering the standard tonometer such as Goldmann applanation tonometer. The simulation is repeated with Goldmann Applanation tonometer of 3.06 mm diameter, at 20 mmHg IOP of eye and by varying the plunging depth of the cornea from 0.3 to 0.7 mm in the steps of 0.1 mm along the direction towards retina. The reaction force experienced by the Goldmann applanation tonometer for providing the plunging depth of cornea from 0.3 to 0.7 mm in steps of 0.1 mm was found out to be 0.028956 N, 0.031776 N, 0.034597 N, 0.037417 N and 0.040237 N, respectively. The results are very close to the observations obtained with the tonometer of 1.7 mm diameter. The results prove that the force of applanation on cornea depends on the IOP of eye and the area under the influence of force of applanation and is independent of the dimensions of the tonometer. Also, the force of applanation on cornea varies linearly with respect to the deformation of cornea.

The limitation of the study is that the simulation approach has not considered the flow of aqueous humor in the anterior chamber of the eye to develop the required pressure in the eye.

### Validation

**Fig. 4:**
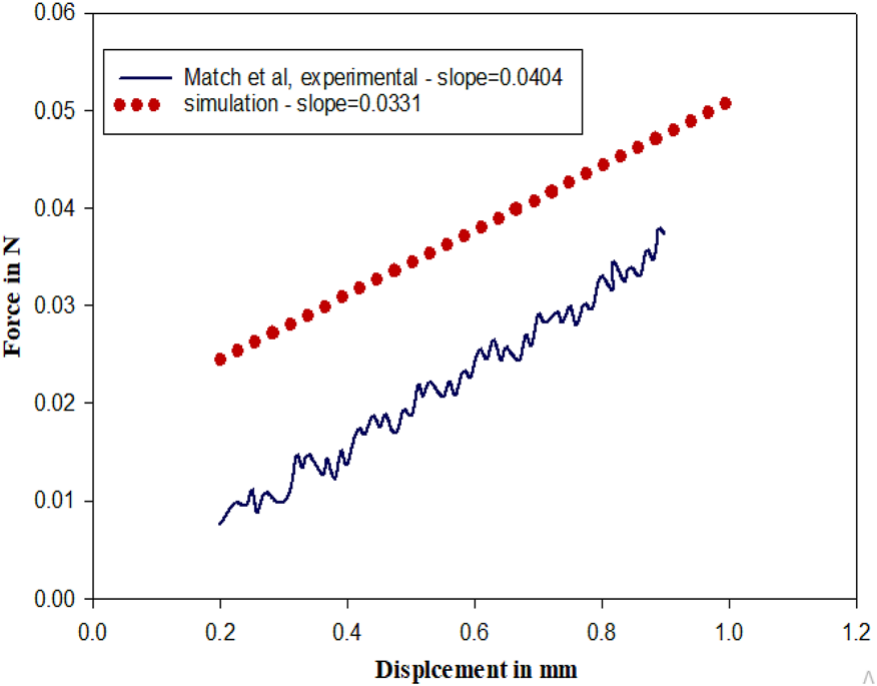
Validation plot

For the validation of the eye model and simulation results, the experimental results from [32] was considered. The experiment was performed by [32], where corneal indentation device is indented on the eye results in displacement over the surface of cornea. Similarly, in simulation approach, plunger of the tonometer applies fixed displacement on the surface of eye and the corresponding force output is observed. The experimental data and the simulation data are compared in Fig. 4. The slope of the data is compared to determine the error. The plot does not lie on each other because of the material properties of tonometer and properties of the eye model will differ from actual eye and indentation device. The plunger used in the experimental study has a diameter of 20 mm, hence the simulation plunger was altered to 2 mm diameter. The average error is 20.055%.

## Conclusion

The outcome of the research is that a maximum force of up to 0.045 N will be applied on the cornea during tonometry with a standard tonometer while the IOP is in the normal range. The analysis has also resulted in quantifying the stress to be 0.2 MPa on the cornea at the junction during tonometry while IOP is in the normal range. The plot of the amount of deformation on the entire surface of the cornea and sclera are measured accurately while performing tonometry. The stress experienced by the cornea at the junction of cornea and tonometer is assessed in the course of tonometry.

In the present study, the force on the cornea is evaluated based on the size of the probe and the amount of deformation of the cornea. In the future may be utilized for the design of new technology development of tonometer or can also be implemented as an additional constituent in any working tonometer. The results can also be utilized as feedback to determine the amount of force applied on the cornea in all the clinical eye testing procedures and if it is increased can act accordingly. In the future, the circulation of aqueous humor in the anterior chamber could be considered for the accurate analysis of the simulation work.

## Data Availability

This is a simulation-based study. The dimensions of the eye during the FEM-based design are referred to from the articles and are cited in the reference list [17–25].
